# Swing Boat: Inducing and Recording Locomotor Activity in a *Drosophila melanogaster* Model of Alzheimer’s Disease

**DOI:** 10.3389/fnbeh.2017.00159

**Published:** 2017-08-30

**Authors:** Johannes Berlandi, Fang-Ju Lin, Oliver Ambrée, Dirk Rieger, Werner Paulus, Astrid Jeibmann

**Affiliations:** ^1^Institute of Neuropathology, University Hospital Münster Münster, Germany; ^2^Department of Biology, Coastal Carolina University Conway, SC, United States; ^3^Department of Psychiatry, University Hospital Münster Münster, Germany; ^4^Department of Behavioral Biology, University of Osnabrück Osnabrück, Germany; ^5^Neurobiology and Genetics, Theodor-Boveri Institute, Biocenter, University of Würzburg Würzburg, Germany

**Keywords:** *Drosophila*, exercise, activity induction, life span, sleep fragmentation, Alzheimer’s disease

## Abstract

Recent studies indicate that physical activity can slow down progression of neurodegeneration in humans. To date, automated ways to induce activity have been predominantly described in rodent models. To study the impact of activity on behavior and survival in adult *Drosophila melanogaster*, we aimed to develop a rotating tube device “swing boat” which is capable of monitoring activity and sleep patterns as well as survival rates of flies. For the purpose of a first application, we tested our device on a transgenic fly model of Alzheimer’s disease (AD). Activity of flies was recorded in a climate chamber using the *Drosophila* Activity Monitoring (DAM) System connected to data acquisition software. Locomotor activity was induced by a rotating tube device “swing boat” by repetitively tilting the tubes for 30 min per day. A non-exercising group of flies was used as control and activity and sleep patterns were obtained. The GAL4-/UAS system was used to drive pan-neuronal expression of human Aβ42 in flies. Immunohistochemical stainings for Aβ42 were performed on paraffin sections of adult fly brains. Daily rotation of the fly tubes evoked a pronounced peak of activity during the 30 min exercise period. Pan-neuronal expression of human Aβ42 in flies caused abnormalities in locomotor activity, reduction of life span and elevated sleep fragmentation in comparison to wild type flies. Furthermore, the formation of amyloid accumulations was observed in the adult fly brain. Gently induced activity over 12 days did not evoke prominent effects in wild type flies but resulted in prolongation of median survival time by 7 days (32.6%) in Aβ42-expressing flies. Additionally, restoration of abnormally decreased night time sleep (10%) and reduced sleep fragmentation (28%) were observed compared to non-exercising Aβ42-expressing flies. On a structural level no prominent effects regarding prevalence of amyloid aggregations and *Aβ42* RNA expression were detected following activity induction. The rotating tube device successfully induced activity in flies shown by quantitative activity analysis. Our setup enabled quantitative analysis of activity and sleep patterns as well as of survival rates. Induced activity in a *Drosophila* model of Alzheimer’s disease improved survival and ameliorated sleep phenotypes.

## Introduction

Physical activity has been shown to influence various metabolic, developmental and behavioral processes (Braam et al., 2013; Hawley et al., 2014; Blundell et al., 2015; Fernandes et al., 2015). Recent studies provided additional indication that exercise decreases the risk and slows down the progression of neurodegeneration in humans (Intlekofer and Cotman, 2013). Animal studies mainly performed in rodents have been conducted to explore the mechanisms involved in the beneficial effects of voluntary and forced exercise (Richter et al., 2008; Rao et al., 2015; Tapia-Rojas et al., 2015). In contrast to running wheels or tread mills used in rodent models, there is no comparable way described to investigate the influence of exercise in *Drosophila*. In this study, we developed a rotating tube device that induces activity while automatically analyzing locomotor behavior, sleep parameters and survival rates.

To test our setup, we investigated the effect of physical exercise on Aβ42-expressing flies, which have been shown to model aspects of Alzheimer’s disease (AD). In the past, several attempts have been made to study effects of transgenic expression of Aβ peptides or APP processing enzymes in *Drosophila* (Ye and Fortini, 1999; Greeve et al., 2004; Iijima et al., 2004; Cao et al., 2008; Ju et al., 2014). Finelli et al. (2004) used the binary GAL4-UAS (Brand and Perrimon, 1993) system to achieve expression of human Aβ42 peptides in *Drosophila*. This approach to model AD in the fruit fly revealed dose-dependent phenotypes distinguishable by shortened life span and accumulations of insoluble Aβ42 peptides. We aimed to examine whether moderate physical activity induced by “swing boat” device can contribute to attenuation of neurodegenerative symptoms on behavioral and structural level in a *Drosophila* model of AD.

The idea to induce locomotor activity in *Drosophila* to study the effect on wild type and pathological conditions is longstanding, Piazza et al. (2009) were the first to apply an exercise regimen and measured locomotor behavior in flies. They designed the “power tower” in which vials are dropped vertically, forcing the flies to the ground. In an immediate response, flies start to climb to the top of the vial due to their tendency to fight gravity (negative geotaxis) thereby continuous climbing (exercise) is induced. In order to quantify behavior in *Drosophila* (social behavior, learning and locomotor activity), a number of assays have been established (Branson et al., 2009; Dankert et al., 2009; Kabra et al., 2013). To evaluate the effect of exercise, Piazza’s (Piazza et al., 2009) group chose the commonly used negative geotaxis assay, which is a simple but relatively time consuming method to obtain and compare locomotor performance of adult fruit flies. Flies are observed during movement induction and maximum height achieved by vertical climbing is recorded and analyzed for each individual animal.

Interestingly, flies placed on the power tower showed significantly improved mobility, compared to stationary control flies that exhibit age-related locomotor impairment (Gargano et al., 2005; Piazza et al., 2009; Tinkerhess et al., 2012). Although exercise induction by repeatedly force-tapping seems effective, however, physical traumatization of tested animals should be taken into consideration. Further limitations of the follow-up climbing assay comprise the requirement of anesthesia prior to each experiment in order to place the animals into the test vial, the need for an experimenter which limits any high-throughput process and finally the difficulty to standardize the tapping down of the test vials.

Recently, Mendez et al. (2016) designed a rotational device which offers some advantages compared to traditional climbing assays. Locomotor activity is induced by rotating vials containing groups of flies. Thus, a large number of animals can undergo exercise induction at the same time without manually tapping food vials. In line with previous studies exercising flies showed increased climbing ability (Piazza et al., 2009) and furthermore reduced stored triglycerides, glycogens and body weight.

Nevertheless, the set up requires transferring anesthetized flies from food vials into exercise tubes prior to each exercise unit and does not include any data acquisition during running experiments. To address the effect of physical activation on locomotor performance, additionally negative geotaxis assays have to be performed subsequently to activity induction which extents duration and limits throughput of experiments. Further it is to consider that the response of adult fruit flies to rather gentle stimuli evoking locomotor activity can be highly individual and is also dependent on group dynamics (Ramdya et al., 2017). Therefore, evaluation of the efficiency of activity induction protocols can be problematic in case of group housing and is susceptible to social effects within the group.

Our device, deemed the “swing boat”, is a combination of a gentle rocker of our own design and an existing commercially available *Drosophila* Activity Monitoring (DAM) System. The rocker simulates a pendulum’s motion, alternating the top and bottom ends of the fly tubes to induce walking activity for 30 min per day. Thus, it has the advantage over previous approaches for it minimizes the stress derived from blunt force and offers a robust methodology to study the effects of exercise in individual animals. In addition, the DAM system enables the experimenter to measure locomotor activity before, during and after exercise without manipulating the flies as the frequently used climbing assays (i.e., negative geotaxis assays), therefore allowing objective data collection to study behavior and survival of adult fruit flies.

## Materials and Methods

### Fly Husbandry

Aβ42-expressing flies (*UAS-Aβ42^H29.3^/CyO*) were provided by Konsolaki (2013). Flies were kept at 25°C and 60% humidity. Flies were crossed against *elav*^c155^-GAL4 (obtained from Bloomington *Drosophila* Stock Center, Bloomington, IN, USA) to activate the UAS-construct pan-neuronally. To control for Aβ42-dependent effects *elav*^c155^-GAL4, *UAS-Aβ42/+* (crossed to Oregon-R wild type strain) and wild type (Oregon-R) flies were analyzed. For all experiments 1 day old male flies were collected immediately after hatching.

### Preparation of Activity Tubes

Food for DAMs (Trikinetics Inc., Waltham, MA, USA) consisted of a mixture of 1% Agar Agar (Kobe I (Carl Roth GmbH and Co. KG, Karlsruhe, Germany) and 5% D (+) sucrose in deionized water, which were boiled first and used to fill up to one-third length of glass tubes (5 mm × 65 mm) specifically designed for DAMs. After the sucrose/agar mixture solidified, glass tubes were sealed with warm paraffin to prevent dehydration.

### Swing Boat Rotating Tube Device

**Figure 1:**
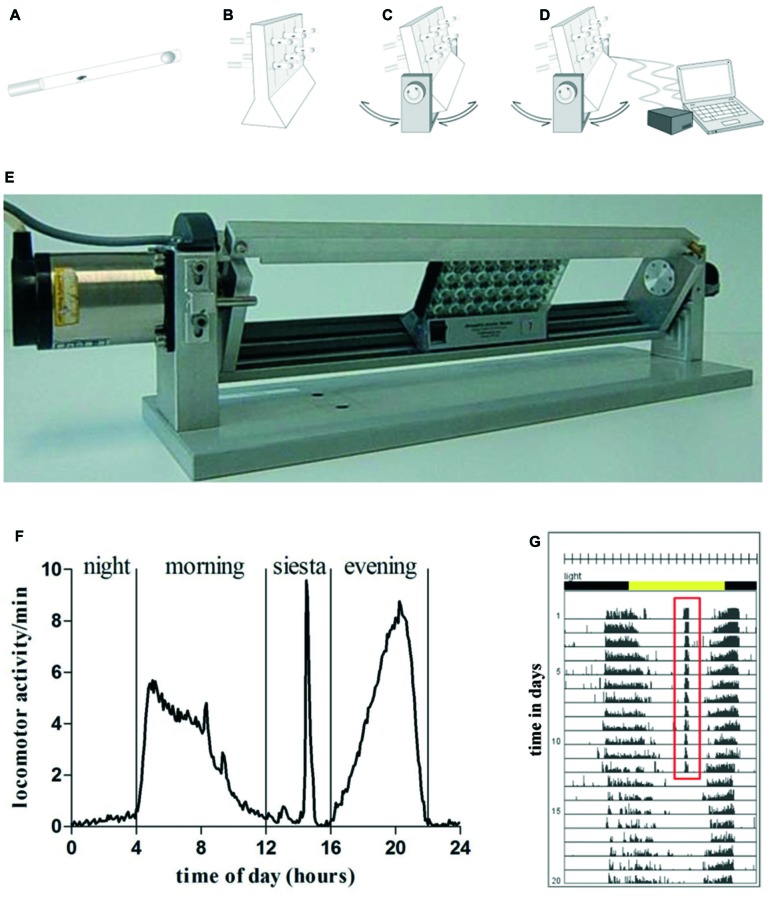
Graphic depiction of swing boat device **(A–D)**. Flies were inserted into a glass tube, that was closed with food on one side and with foam material on the other side **(A)**. Glass tubes were put into *Drosophila* activity monitoring (DAM) **(B)** that was fixed on the swing boat device. During daily 30 min training period the tubes alternately turned 30° to each side so that the top and bottom ends of the fly tubes rotated and induced a negative geotaxis response **(C)**. The device was connected to the steering unit which allowed defining speed, turning angle und frequency of turns **(D)**. A single swing boat device that can carry up to three DAMs **(E)**. **(F)** Daily activity of wild type fly undergoing 30 min of daily activity induction. One full day is divided in four activity episodes: night, morning, siesta and evening. During the time of siesta sleep when exercise units were given the animal shows pronounced locomotor activity. **(G)** Actogram of exemplary wild type fly undergoing 12 days of activity induction. Training period is represented by days 1–12, the post-training period comprises days 13–20. Total number of movements was summed at the end of each 5-min interval over the entire time of experiment. Each day (rows) was visualized in 288 bars each representing one 5-min interval. A 12:12 h light dark cycle (LD12:12) is shown by environmental bars (lights on 8 am: ZT0, yellow color; lights off: ZT12, gray color). Red (ZT7) marks activity at this time.

Fly locomotor activity was induced by the swing boat device constructed for this purpose (Figure 1). It is composed of a metal holder with a movable metal swing. DAMs (Model DAM2, Trikinetics Inc., Waltham, MA, USA; up to three) were placed on the swing and fastened with a metal strap to prevent sliding. The device was placed in a climate chamber (darkened glass aquarium: 80 × 40 × 35 cm^3^) providing steady environmental conditions during the experiment. Temperature and humidity were continuously controlled by a climate control system (Dohse Aquaristik KG, Grafschaft-Gelsdorf, Germany) and kept at 25°C and 60% relative humidity using external humidifiers and heating cables (Lucky Reptile Super Fog Nano, Lucky Reptile, Waldkirch, Germany), respectively. Animals were entrained in a 12:12 h light dark cycle (LD12:12) using a time switch, lights on at 8 am. A two phase hybrid step motor (Phytron, ZSS57.200, Phytron GmbH, Gröbenzell, Germany) was used to invoke the DAM monitors that hold the test tubes. The motor was connected to a micro controller board (Arduino Uno, Konrad Electronics, Hirschau, Germany), which allowed digital adjustment of the following conditions: displacement angle, velocity as well as time point and duration of activity induction.

### Activity Induction using Swing Boat Device

Flies were positioned in DAMs on the platform and habituated for 1 day before the exercise phase started. The training phase lasted 12 days. Swing boat rotation started 7 h after turning the lights on (*Zeitgeber* time, ZT7) and the flies were moved for 30 min in repetitive cycles with a displacement angle of *φ* = 30°, angular velocity *ω* = 30°/s and three turns per minute. Rocker was first tilted clockwise, pausing for 17 s, then followed by tilting at 30° counter-clockwise (Figures 1C–E). Flies were exposed to a total number of 90 stimuli per daily exercise unit. Subsequent to the exercise phase flies were kept under the same conditions for the entire life span. Trikinetics data acquisition software (DAMSystem308, Trikinetics, Waltham, MA, USA) was used to save activity data as channel counts per time period during the whole experiment.

### Monitoring Locomotor Activity of Adult

#### Drosophila

The DAM System enables to acquire and compare activity data of different genotypes and treatment groups (exercise vs. non-exercise) with a large number of animals. Up to 32 flies can be placed in one DAM each into an individual channel equipped with an infrared light beam to detect movement when interrupted. Considering 5 min of inactivity as sleep and immobility more than 24 h as death event, the obtained data can be processed to quantify locomotor activity, sleep duration and survival of the flies. Apart from simply quantifying sleep duration, resting phases can be further characterized by the length of individual sleep bouts. Long-term sleep includes sleep bouts longer than 1 h, whereas short-term sleep considers only fragmented sleep episodes of maximum 60 min inactivity. Trikinetics data acquisition software (DAMSystem308, Trikinetics Inc.) saves activity data as channel counts per time period. ImageJ software (available: http://imagej.nih.gov/ij/) combined with the freely available ActogramJ plug-in (v0.9; Schmid et al., 2011) was used to draw periodic actograms for each individual fly. Calculation and statistical evaluation of raw data was carried out using Microsoft Excel and Graphpad Prism (Graphpad Software, La Jolla, CA, USA). All analyzed activity and sleep parameters were calculated for each day of the experiment as average from data of all living animals at this time and afterwards displayed over the duration of the experiment (up to 20 days).

### RNA Isolation and cDNA Synthesis

Twenty heads of exercising and 20 heads of non-exercising flies were collected at day 16 of the experiment (2 days after completing exercise phase) for RNA isolation. Each experiment was carried out in biological triplicates. Maxwell^®^ 16 LEV simplyRNA Tissue Kit (Promega GmbH, Mannheim, Germany) was used according to manufacturer instructions. Heads were kept in 200 μl of homogenization solution and then carefully ground with a sterile pestle for 2 min, then lysis buffer was added. Implen Nanophotometer P300 (Implen GmbH, Munich, Germany) was used to measure RNA concentrations. The High Capacity cDNA Reverse Transcriptase Kit (Applied Biosystems, Darmstadt, Germany) was used to obtain cDNA from purified RNA samples. Reverse transcription reaction was carried out in MJ Thermal Cycler PTC 200 (MJ Research Inc., Ramsey, MN, USA).

### Quantitative Real-Time PCR

*Aβ42* mRNA was quantified with primers and a probe designed for this purpose (Eurofins MWG), (Aβ42 probe (10 pmol/μl): FAM-CGCTTGGGTCCTGCCTCCTGG-TAM; Aβ42 forward: GAGACTTTGCATCTGGCTGCTA; Aβ42 reverse: TGCGTCTGCCTGCACTGTA). Expression levels of house-keeping gene dGAPDH were additionally detected for normalization (Assay ID: Dm01843827_s1). Quantitative gene expression assay was performed using the Step One PlusTM Real-Time PCR System (Applied Biosystems). The Step One Software 2.2 (Applied Biosystems) was required to generate and analyze the data using the ∆∆Ct-method. All experiments were conducted in three biological as well as three technical replicates.

### Immunohistochemistry

Five micrometer thin paraffin sections of 20 days old fly brains (exercise vs. non-exercise) were deparaffinized, rehydrated and washed in distilled H_2_O. For antigen retrieval, slides were pretreated with formic acid (98%–100%, 3 min). Anti-amyloid beta (M872, mouse monoclonal, 1:100, DAKO, Glostrup, Denmark) was used. After washes in PBT, the slides were incubated with a biotinylated goat anti-rabbit secondary antibody (E0432; 1:500 dilution; DAKO, Glostrup, Denmark) for 45 min at room temperature after incubation with the ABC kit (SK6100; Vectastain avidin-biotin complex-horseradish peroxidase (ABC-HRP); Vector Laboratories, Burlingame, CA, USA) for 45 min after washing in PBT. The signal was developed using a 3,3-diaminobenzidine (DAB) substrate kit (SK4100; Vector Laboratories), and the sections were counterstained with hematoxylin (DAKO, Glostrup, Denmark A/S; hematoxylin S3301, 5 min). For negative controls, sections were stained as described above using only the secondary antibody.

### Microscopy and Image Processing

For image acquisition of brain sections an Olympus microscope BX51 (Olympus GmbH, Hamburg, Germany) and the Cell R software (Olympus GmbH) was used. Processing and alignment of image data were executed using Adobe Photoshop CS5 (Adobe Systems Software Ireland Ltd., Dublin, Ireland). For Quantification of Aβ42 positive structures ImageJ software (Cell counter plugin^1^) was used.

### Statistics

Data are expressed as means ± SEM. Statistical significance was determined by two tailed Student’s *t*-test for DAM data (sleep, fragmented sleep, activity) and Mann-Whitney-U test (quantitative real-time PCR, immunohistochemistry). *P*-values smaller than 0.05 are marked *, *p*-values < 0.01 ** and *p-values* < 0.001 ***. Survival data were analyzed using Log rank (Mantel-Cox) test. Raw data were processed using Microsoft Excel. Statistics were calculated and graphs were drawn using Graphpad Prism (Graphpad Software, La Jolla, CA, USA).

## Results

### Swing Boat Device can Induce Activity in Flies

**Figure 2:**
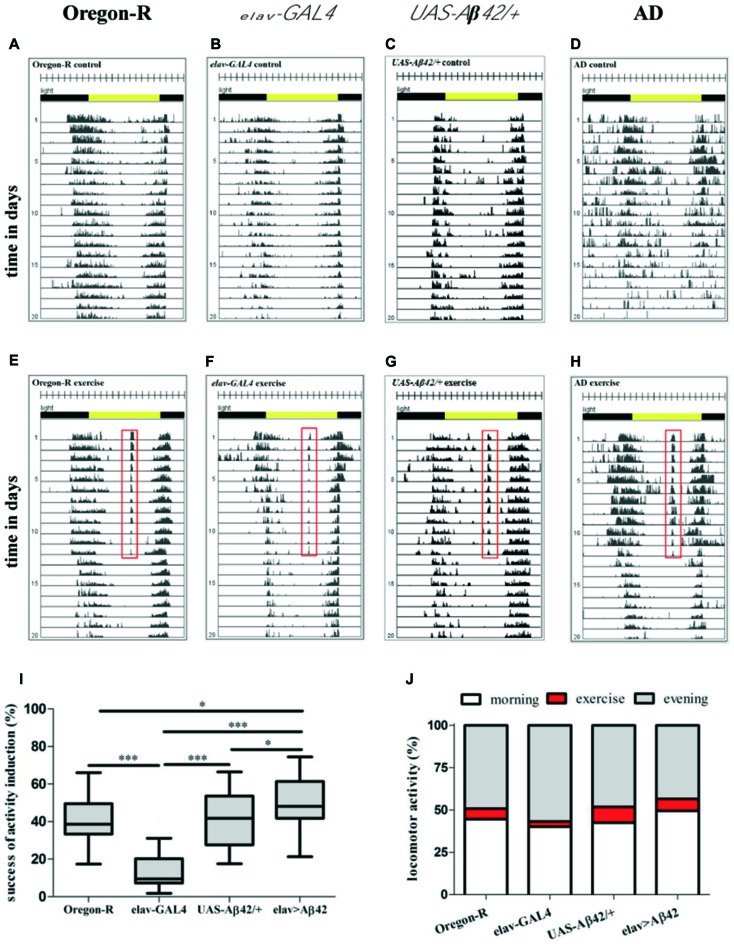
Representative actograms of individual animals of non-exercising **(A–D)** and exercising **(E–H)** wild type (Oregon-R), *elav-GAL4, UAS-Aβ42/+* and Alzheimer’s disease (AD) flies. For exercising flies the training period is represented by days 1–12, the post-training period comprises days 13–20. Total number of movements was summed at the end of each 5-min interval over the entire time of experiment. Each day (rows) was visualized in 288 bars each representing one 5-min interval. A 12:12 h light dark cycle (LD12:12) is shown by environmental bars (lights on 8 am: ZT0, yellow color; lights off: ZT12, gray color). Red frame in actograms of flies exposed to 30 min of daily exercise (ZT7) marks activity at this time **(E–H)**. **(I)** Box plots (Whisker’s: Tukey; median and percentile (25–75%)) display percentage activity induction success of AD flies and control groups. For quantification of activity induction DAMs were loaded with 32 animals per genotype in the beginning of the experiment. Success of activity induction (%) during 30 min of daily exercise was assessed for AD flies (*elav-GAL4 > UAS-Aβ42*) and control strains (Oregon-R*, elav-GAL4, UAS-Aβ42/+*). Exercise success was calculated for each day of 12 days of activity induction for each animal to obtain average success for each genotype. During 30 min daily exercise flies were exposed to 90 stimuli (3 per min) by rotatory tilting the rocker holding DAMs by an angle of *φ* = 30°. Success of activity induction amounted to 38.62% (median; 25% percentile: 33.36%; 75% percentile: 49.55%) for Oregon-R flies, 9.44% (median; 25% percentile: 7.32%; 75% percentile: 20.19%) for *elav-GAL4* strain, 41.76% (median; 25% percentile: 27.59%; 75% percentile: 53.61%) for *UAS-Aβ42/+* flies and 48.14% (median; 25% percentile: 41.78%; 75% percentile: 61.41%) for AD flies. AD flies reacted significantly stronger to rocker motion compared to Oregon-R (*p < 0.05), *elav-GAL4* (***p < 0.001) and *UAS-Aβ42/+* flies (*p < 0.05). Response to activity induction of *elav-GAL4* flies was significantly lower compared to Oregon-R, *UAS-Aβ42/+* and *elav-GAL4* strains (***p < 0.001). **(J)** Induced locomotor activity was quantified in relation to overall activity (morning peak and evening peak) during a full day. Exercising constituted 6.3% of overall locomotor activity in Oregon-R group, 3.1% of *elav-GAL4*, 9.4% in *UAS-Aβ42/+* group and 7.1% in AD flies.

To test the new device, we designed an experiment to investigate the effect of physical exercise on behavior (locomotor activity and sleep) and survival in Aβ42-expressing flies which have been shown to model aspects of Alzheimer’s disease such as Aβ deposition and reduced survival. Newly hatched male flies of *elav-GAL4 > UAS-Aβ42* (AD flies) and control flies (Oregon-R, *elav-GAL, UAS-Aβ42/+*) were placed individually into glass tubes (Figure 1A). DAM monitors (Figure 1B) were fastened on the device (Figure 1E). Baseline recording of locomotor activity without rocking was carried out for 24 h before the experiment started. After that, daily exercise for 30 min started at ZT7, which included repetitive cycles of rocking motions at 30°. Control flies (non-exercising group) were kept in another chamber with identical light-dark cycle, temperature and humidity. After 12 days of exercise were given to the experimental groups, the rocker was turned off and the flies remained stationary over the entire life span. Data were recorded by DAM System software and graphed. A wild type fly exposed to 30 min of activity induction during the time of siesta sleep shows elevated activity at this time (Figures 1F,G). Comparisons of representative actograms of non-exercising (Figures 2A–D) and exercising (Figures 2E–H) flies revealed pronounced activity peaks for flies undergoing exercise induction at this time. Actograms of Aβ42-expressing flies (Figure 2D) display frequent events of spiking activity when lights are turned off (ZT12-ZT0). Events of spiking activity were less abundant in Oregon-R, *elav-GAL4* and *UAS-Aβ42/+* control groups (Figures 2A–C) as well as in exercising AD flies (Figure 2H).

**Figure 3:**
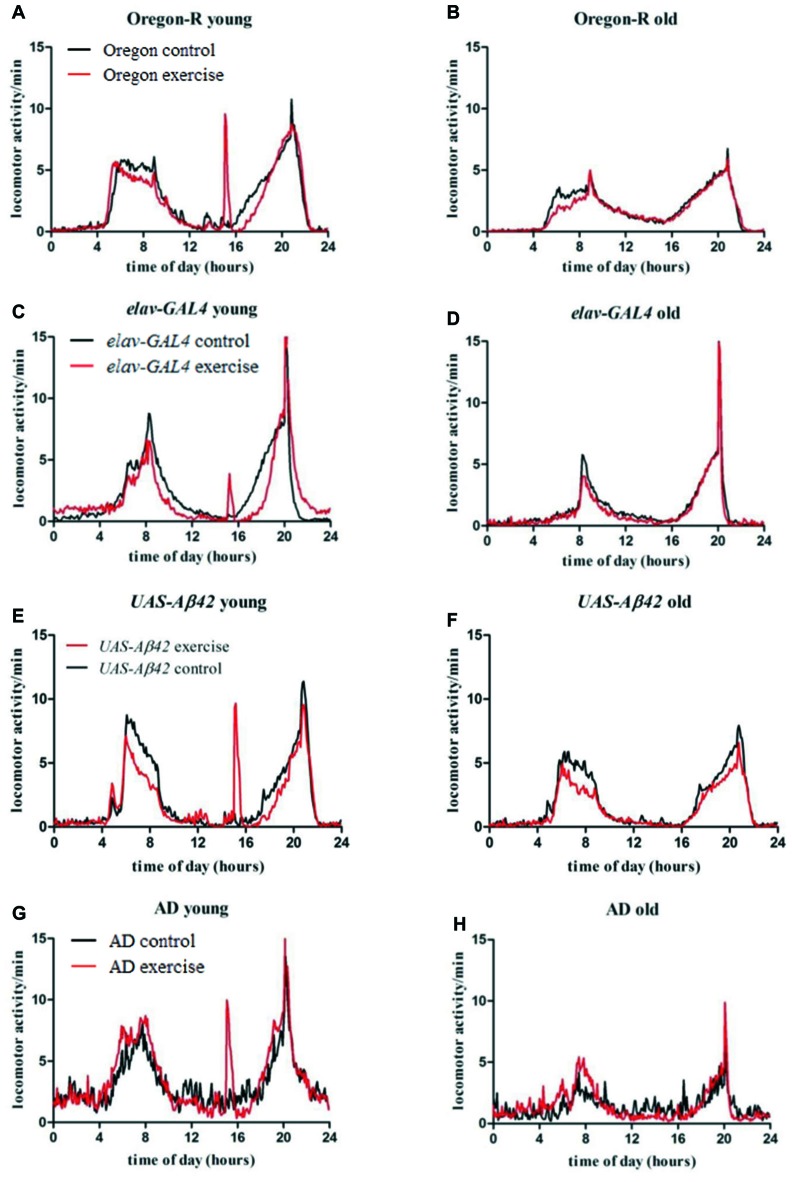
Average locomotor activity (activity counts per 5 min) of 32 flies per genotype was measured over 20 days for exercising (red line) and stationary animals (black line) in a 12:12 h light dark cycle (LD12:12; lights on 8 am: ZT0). Data are plotted over time of the day to visualize average activity in the morning (4 am–12 am), evening (4 pm–10 pm) as well as during activity induction (3 pm–3.30 pm) and siesta sleep (12 am–4 pm), respectively. Left figures **(A,C,E,G)** show activity of young flies during activity induction (day 1–12), right figures **(B,D,F,H)** display activity of old flies in the post-training phase (day 13–20).

Young stationary Oregon-R flies (Figure 3A, black line) show characteristic activity peaks in the morning and evening separated by siesta sleep. Exercising evoked a third distinct activity peak in wild type flies (Figure 3A, red line) followed by recovering of siesta sleep subsequently to activity induction. Morning activity peak is less pronounced compared to non-exercising control flies. As observed in young Oregon-R group, morning activity is also decreased in post-exercise phase (Figure 3B, red line).

Stationary *elav-GAL4* and *UAS-Aβ42/+* flies show similar activity distribution compared to Oregon-R strain and are mostly active in the morning and evening (Figures 3C–F, black line). In young as well as in old *elav-GAL4* flies evening activity peak reaches a higher maximum compared to wild type control (Figures 3C,D, black line). As observed in exercising wild type flies, decreased locomotor activity can be also observed in *elav-GAL4* and* UAS-Aβ42/+* flies, subsequently to activity induction and in the morning (Figures 3C,E, red line) compared to non-exercising controls. Exercising *elav-GAL4* flies display a noticeably lower response to activity induction than Oregon-R and *UAS-Aβ42/+* control groups.

Non-exercising young AD flies show typical activity peaks in the morning and evening (Figure 3G, black line). Overall daily locomotor activity can be distinguished from control groups by frequent arrhythmic spikes in locomotor activity causing activity fragmentation during night and siesta sleep. Decline in locomotor activity in old stationary AD is more pronounced compared to control groups (Figure 3H, black line). Exercising AD flies display a strong response to activity induction and less spiking activity during siesta sleep (Figure 3G, red line). In contrast to exercising Oregon-R, *elav-GAL4* and *UAS-Aβ42/+* control flies, no resting (decreased locomotor activity) can be observed after exercising. Furthermore, in old AD flies activity was higher in the morning compared to stationary group (Figure 3H, red line) which was not observed in GAL4, UAS or wild type control flies.

To verify the efficiency of activity induction, the number of movements during 30 min of exercise was summed up. One hundred percent individual exercise success was defined as detecting locomotor activity upon each single rotation of the tube device during 90 rotations over a 30-min period. Activity induction was most successful in AD flies (48.14%), which responded to almost every second activity impulse (tube rotation; Figure 2I). Response to activity induction was significantly higher in AD flies in comparison to Oregon-R (*p < 0.05), *elav-GAL4* (***p < 0.001) and *UAS-Aβ42/+* (*p < 0.05) controls. Oregon-R strain reacted to 38.62% and *UAS-Aβ42/+* flies to 41.76% of activity inductions, displaying higher exercise success than *elav-GAL4* flies (***p < 0.001).* Elav-GAL4* flies poorly responded to activity induction, with only 9.44% of tube rotations provoking locomotor activity.

Overall locomotor activity (monitor counts in the morning and evening) was compared to activity during exercise induction to quantify the share of forced locomotor activity in relation to voluntary daily activity (Figure 2J). Exercising constituted 7.1% of overall monitor counts in AD flies, 6.3% in Oregon-R group and 9.4% in *UAS-Aβ42/+* flies. In exercising *elav-GAL4* flies only 3.1% of daily locomotor activity was induced by exercising.

Whereas exercise units provoked distinct activity peaks during the time of activity induction as well as following resting phases, the overall locomotor activity was not altered in Oregon-R strain and AD flies (Supplementary Figures S2C, S3A). Average locomotor activity of *UAS-Aβ42/+* (day 1–20) and *elav-GAL4* (day13–20) flies was decreased in exercising groups (Supplementary Figures S2A,B).

#### Exercising during Siesta Sleep Induces Recovering in Wild Type Flies

**Figure 4:**
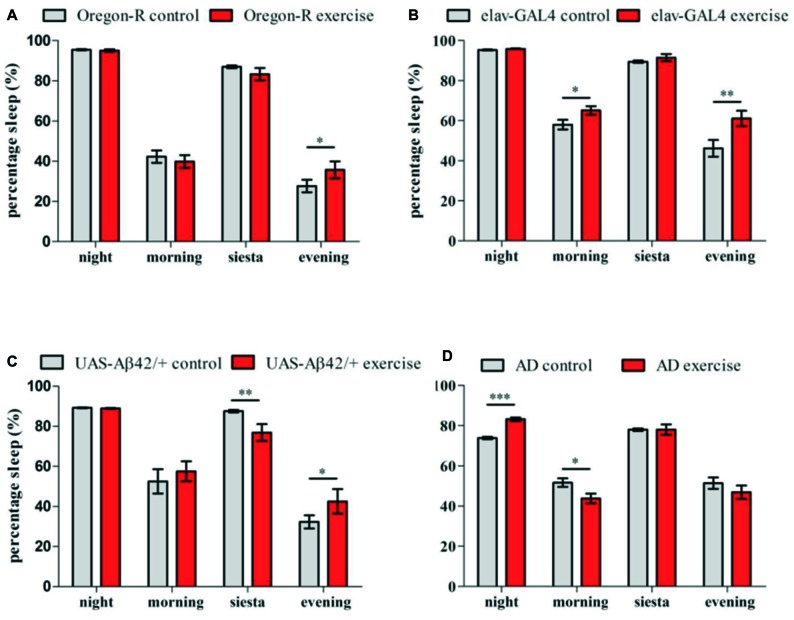
Sleep (5 min of inactivity) was quantified for 32 animals per genotype over 12 days of activity induction and compared to stationary controls (LD12:12, lights on 8 am:ZT0) **(A–D)**. Sleep episodes were categorized into night sleep (22 pm–4 am) and siesta sleep (12 am–4 pm) as well as resting phases during morning (4 am–12 am) and evening activity (4 pm–10 pm). Bar graphs show percentage of time spent sleeping during the night, morning, siesta and evening of exercising flies in comparison to stationary controls. **(A)** Exercising Oregon-R flies spent more time sleeping during the evening compared to non-exercising control (*p < 0.05). **(B)** *Elav-GAL4* flies undergoing activity induction displayed more resting phases than control group in the morning (*p < 0.05) and in the evening (**p < 0.01). **(C)** *UAS-Aβ42/+* flies showed less sleep during activity induction (*p < 0.05) and more activity in the evening (**p < 0.05). **(D)** In exercising AD flies significantly more sleep was measured during the night (***p < 0.001) followed by less resting phases in the morning (*p < 0.05).

Oregon-R, *elav-GAL4* as well as *UAS-Aβ42/+* strain displayed decreased locomotor activity subsequently to activity induction, indicating that animals are recovering sleep after exercising during siesta. To address whether exercising can affect sleep and resting phases during the day, episodes of inactivity were quantified in the night, morning, during siesta sleep and evening over a period of 12 days of activity induction (Figures 4A–D).

Quantification of resting phases (sleep) in the evening revealed elevated sleep levels for exercising Oregon-R (*p < 0.05; Figure 4A), *elav-GAL4* (**p < 0.01; Figure 4B) and *UAS-Aβ42/+* flies (*p < 0.05; Figure 4C). Additionally, *elav-GAL4* flies spent more time sleeping in the morning (*p < 0.05) and *UAS-Aβ42/+* flies displayed less sleep during exercise units (**p < 0.01) compared to non-exercising controls. In contrast to all control groups, exercising AD flies did not show reinforced resting in the evening after exercise units were given (Figure 4D). Instead, Aβ42-expressing flies undergoing activity induction had increased night time sleep (***p < 0.001) and displayed fewer resting phases in the morning (*p < 0.05). Non-exercising AD flies spend less time sleeping in the night compared to stationary Oregon-R (***p < 0.001) *elav-GAL4* (***p < 0.001) and *UAS-Aβ42/+* group (***p < 0.001; Figures 4A–D).

### Aβ42-Expressing Flies Display Abnormalities in Activity and Sleep

**Figure 5:**
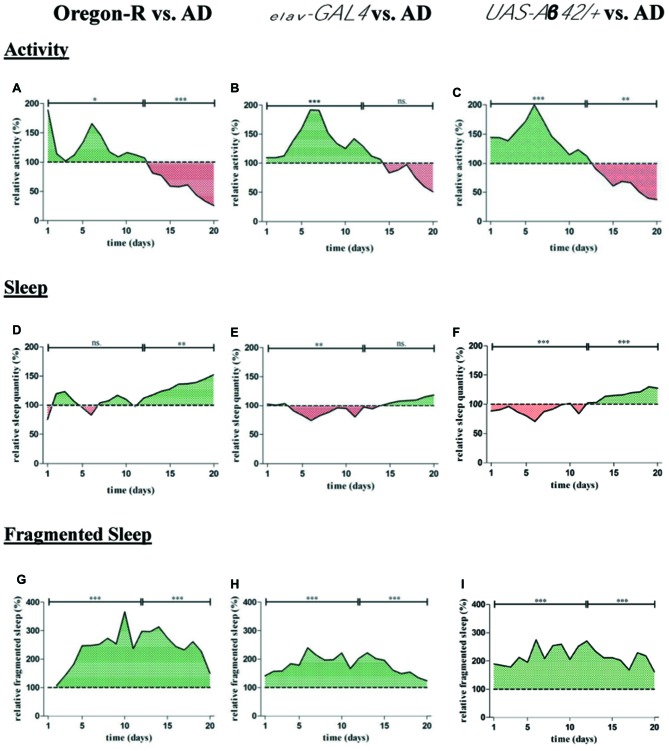
For quantification of average locomotor activity, sleep as well as fragmented sleep, DAMs were loaded with 32 animals per genotype in the beginning of the experiment. **(A–C)** Relative activity of AD flies (*elav-GAL4 > UAS-Aβ42*) was assessed by normalization to Oregon-R* elav-GAL4* and *UAS-Aβ42/+* control. Total number of movement counts were averaged for each day and displayed over a period of 20 days in relation to control groups. Activity of controls is considered as 100%. **(A)** Locomotor activity of AD flies was increased from day 1 to 12 (*p < 0.05) and decreased from day 13 to 20 (***p < 0.001) compared to Oregon-R strain. **(B)** Relative activity of AD flies was increased from day 1 to 12 (***p < 0.01) compared to *elav-GAL4* driver strain. **(C)** Relative activity of AD flies was increased from day 1 to 12 (***p < 0.01) and reduced from day 13 to 20 (**p < 0.001) compared to *UAS-Aβ42/+* control. **(D–F)** Relative sleep quantity of AD flies was assessed by normalization to Oregon-R, *elav-GAL4* and *UAS-Aβ42/+* control. Total sleep duration was averaged for each day and is shown over 20 days of data acquisition in relation to control groups. Sleep duration of controls is considered as 100%. Daily sleep in AD flies was decreased from day 1 to 12 compared to *elav-GAL4* (**p < 0.01) and increased in comparison to Oregon-R control from day 13 to 20 (**p < 0.01). AD flies showed less sleep from day 1 to 12 (***p < 0.001) and elevated daily sleep from day 13 to 20 (***p < 0.001) compared to *UAS-Aβ42/+* flies. **(G–I)** Relative fragmented sleep (short term sleep) of AD flies was assessed by normalization to Oregon-R, *elav-GAL4* and *UAS-Aβ42/+* control.Total short term sleep was averaged for each day and is shown over 20 days of experiment in relation to control groups. Fragmented sleep of controls is considered as 100%. Short term sleep quantity of AD flies was increased over a period of 20 days when compared to Oregon-R (***p < 0.001), *elav-GAL4* (***p < 0.001) and *UAS-Aβ42/+* control flies (***p < 0.001).

Locomotor activity, sleep and episodes of fragmented sleep were chosen as parameters to identify behavioral abnormalities in Aβ42-expressing flies (Figure 5). To measure activity and sleep of AD flies, the DAM monitoring system was used. From day 1–12 activity of AD flies was increased (+24.7%) compared to Oregon-R flies (*p < 0.05) followed by a decline in activity (−55.3%) in 13–20 days old Aβ42-expressing flies (***p < 0.001; Figure 5A). Similar to Oregon-R control also *elav-GAL4* and *UAS-Aβ42/+* flies were less active than AD flies (*elav-GAL4*: −43.6%, ***p < 0.001;* UAS-Aβ42/+*: −46.4, ***p < 0.001) in the first half of the experiment (day 1–12) and showed more activity monitor counts (*elav-GAL4*: +22.3%, *p* > 0.05); *UAS-Aβ42/+*: +38.6%, **p < 0.01) from day 13–20 (Figures 5B,C).

Relative sleep duration of AD flies was decreased from day 1–12 compared to *elav-GAL4* (−11%; **p < 0.01) and *UAS-Aβ42/+* (−10.2%, ***p < 0.001) but not in comparison to Oregon-R group (Figures 5D–F). From day 13–20 AD flies slept significantly more than Oregon-R (+34.7%, **p < 0.01) as well as *UAS-Aβ42/+* flies (+18.2%, ***p < 0.001) and displayed a slight increase in sleep from day 13–20 compared to *elav-GAL4* flies (*p* > 0.05).

Additionally, we found that sleep fragmentation of AD flies was strongly increased over a period of 20 days of data acquisition when compared to both, *elav-GAL4* (+80%; ***p < 0.001) and *UAS-Aβ42/+* (+116.2%; ***p < 0.001) control group as well as in comparison to Oregon-R wild type strain (+158%; ***p < 0.001; Figures 5G–I).

### Exercise Induced Effects in a Fly Model of Alzheimer’s Disease

To address, whether gently induced physical activity can alter phenotypes of human Aβ42-expressing fruit flies, the DAM System in combination with a rotating tube device were used to monitor AD flies undergoing activity induction. After a training protocol consisting of daily exercise over the entire life span of the animals was found to be detrimental for AD flies (Supplementary Figure S1) a protocol of 12 consecutive days of 30 min exercise was designed. Oregon-R wild type flies, e*lav-GAL4* driver strain and *UAS-Aβ42/+* reporter strain (crossed to Oregon-R wild type strain) were used as controls. Non-exercising flies did not show relevant activity during siesta sleep at time ZT7 when exercise units were given (Figures 2A–D, 3A,C,E,G, black line).

**Figure 6:**
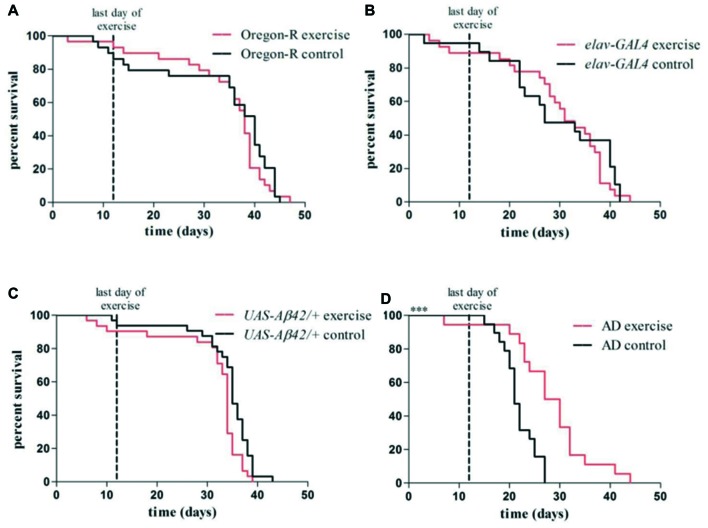
Kaplan–Meier plots show survival rates of exercising flies compared to non-exercising control groups **(A–D)**. Red lines represent survival curves of exercising groups, black lines show survival curves of non-exercising control groups. Vertical line at day 12 marks the last day of the exercise phase. **(A–C)** Exercising in the swing boat device did not significantly affect survival of Oregon-R (40.0 days), *elav-GAL4* (31.0 days) and *UAS-Aβ42/+* (34.0 days) control flies compared to non-exercising controls (Oregon-R: 38.0 days, *elav-GAL4*: 27.0 days, *UAS-Aβ42/+*: 35.0 days). **(D)** Median survival time of exercising AD flies (28.5 days) was significantly longer (***p < 0.001) compared to non-exercising AD flies (21.5 days).

Exercising AD flies survived significantly longer than non-exercising AD flies (median survival: 28.5 vs. 21.5 days, ***p < 0.001; Figure 6D) and showed a similar life span compared to *elav-GAL4* (non-exercise: 27.0; exercise: 31.0) and *UAS-Aβ42/+* (non-exercise: 35.0; exercise: 34.0) control flies. Survival rates of control groups were not affected by undergoing the identical exercise protocol (*p* > 0.05; Figures 6A–C). Oregon-R wild type flies displayed the longest life span of all tested groups (non-exercise: 40.0; exercise: 38.0; Figure 6A).

**Figure 7:**
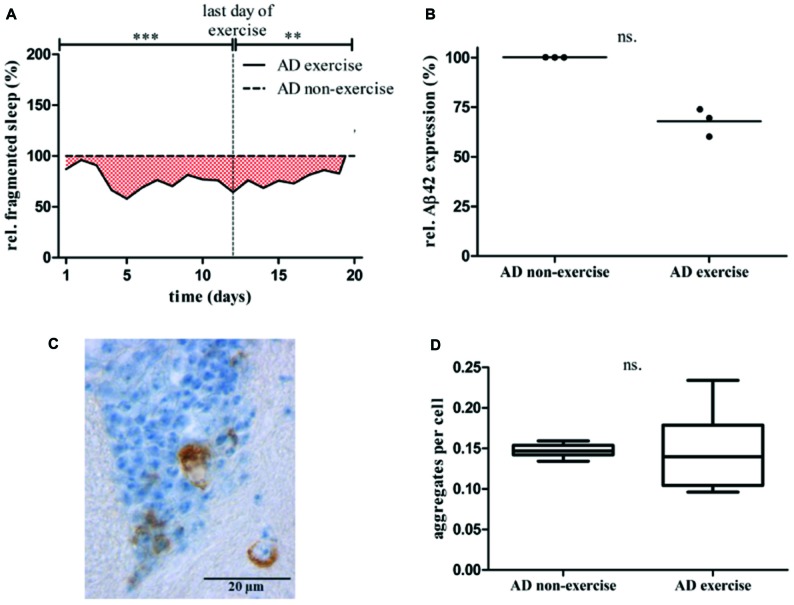
**(A)** Relative fragmented sleep (short term sleep) of exercising AD flies (*elav-GAL4 > UAS-Aβ42*) was assessed by normalization to non-exercising AD control. Total short term sleep for 32 animals per group averaged for each day is shown over 20 days of data acquisition. Short term sleep of non-exercising controls is considered as 100%. Vertical line at day 12 marks the last day of exercise. Fragmented sleep was reduced by 31.6% from day 1 to 12 (***p < 0.001) and 22.2% (**p < 0.01) lower from day 13 to 20 compared to stationary AD control. **(B)** Sixteen days old exercising AD flies show lower *Aβ42* mRNA expression level in comparison to non-exercising AD flies. Three biological triplicates (comprising 20 adult fly heads each) revealed residual *Aβ42* mRNA expression of 67.8% (*p* > 0.05) in 16 days old Aβ42-expressing flies after undergoing 12 days of activity induction. **(C)** Five μm thick paraffin sections of 20 days old pan-neuronally Aβ42-expressing flies were stained using α-Aβ42 antibody (6F/3D). Amyloid-beta staining is shown in brown, nuclear hematoxylin staining in blue (160x). Round and crescent-shaped Aβ42 aggregations in the central brain. **(D)** Aβ42 aggregates were quantified in paraffin sections of exercising and non-exercising AD flies. Brain sections from a representative layer were chosen to quantify Aβ42 containing protein aggregations in adult brains of exercising and non-exercising AD flies (*n* = 6). Paraffin sections of 20 days old flies were stained using α-Aβ42 antibody (6F/3D). Non-exercising AD flies displayed 0.15 ± 0.01 aggregates/cell, exercising AD flies 0.15 ± 0.05 aggregates/cell.

To investigate whether exercising can affect behavioral phenotypes in AD flies quantity of locomotor activity, sleep and fragmented sleep (short term sleep; sleep bouts <60 min) was compared to non-exercising AD control. Whereas overall locomotor activity was not affected by activity induction, quantity of sleep (Supplementary Figure S2F) and in specific episodes of fragmented sleep (Figure 7A, Supplementary Figure S2I) were found to be decreased in exercising AD flies. Non-exercising AD flies displayed in average 314.9 min, whereas exercising AD flies had 246.0 min of short term sleep per day. Altogether short term sleep quantity of exercising AD flies was significantly reduced by 28.0% compared to non-exercising control (***p < 0.001). During activity induction fragmented sleep was decreased by 31.6% (***p < 0.001) and maintained 22.2% (**p < 0.01) lower after completing exercise phase with regard to stationary AD flies.

Twelve days of consecutive activity induction did not affect overall sleep or short term sleep quantity in Oregon-R strain (Supplementary Figures S3B,C) and *UAS-Aβ42* control group (Supplementary Figures S2D,G). Exercising *elav-GAL4* flies showed elevated levels of sleep over the entire experiment (Supplementary Figure S2E) and more fragmented sleep in the post-exercise phase (Supplementary Figure S2H).

To further address the question, whether activity induction affects *Aβ42* mRNA expression or the prevalence of Aβ42-containing deposits in the adult *Drosophila* brain quantitative real-time PCR and immunohistochemical staining were performed. Quantitative real-time PCR was conducted 2 days after completing the exercise protocol to measure level of *Aβ42* mRNA in 16 days old AD flies. Three biological and three technical measurements revealed residual *Aβ42* mRNA expression of 67.8% in exercising AD flies compared to non-exercising AD control (*p* > 0.05; Mann-Whitney-U; Figure 7B). Additionally, Aβ42 was stained (Figure 7C) and aggregates were quantified in paraffin sections of 20 days old exercising and non-exercising AD flies (Figure 7D). Relative quantity of Aβ42 deposits was calculated by counting the cells displaying Aβ42 aggregations, divided by the overall cell number. Quantity of Aβ42 aggregates in exercising and non-exercising AD flies did not differ at an age of 20 days: 0.15 ± 0.01 aggregates per cell were found in exercising AD flies compared to 0.15 ± 0.05 aggregates per cell in non-exercising flies (*p* > 0.05).

## Discussion

This study aimed to establish a novel methodology to gently induce physical activity in *Drosophila* *melanogaster*. The “swing boat” rotating tube device makes use of the innate negative geotaxis in flies and prevents physical harm during activity induction. Coupled with the DAM System it enables objective data acquisition and requires only minimal manual interference during running experiments. Previous approaches to induce physical activity all consist of a harsh tapping of the flies to the bottom of tubes to evoke negative geotaxis. Despite the fact that induced exercise does improve locomotor performance of *Drosophila* (Piazza et al., 2009; Sujkowski et al., 2015), it also raises concern of introducing stress and physical impairment to the animals. Further attempts to induce walking activity by exposing flies to a gentle rotary motion reduce the influence of external stressors but offer no possibility to monitor individual animals and require frequent manual interference with the flies during running experiments as well (Mendez et al., 2016).

In order to exclude subjectivity, we quantified activity using the DAM System which has been predominantly applied to assess circadian rhythms in flies (Pfeiffenberger et al., 2010). The combination of DAM System with swing boat device offers a highly robust alternative to induce exercise by gentle rocking without impairment of tested animals and automated data acquisition over the lifetime of animals. Thus, not only the success of activity induction can be verified but also multiple behavioral parameters such as activity and sleep characteristics can be easily compared for individual animals before, during and after exercise units.

Our results demonstrated in a first application using a fly model of AD and wild type flies that animals displayed a solid response to activity induction, visualized by actograms (Figures 2E–H). We have tried several approaches to induce physical activity in Aβ42-expressing flies and found that beneficial stimulation is dependent on intensity of the induction protocol. Too many exercise units seemed to provoke physiological stress and shortened life span suggesting that excessive exercise may negate any beneficial long-term and/or short-term effects (Supplementary Figure S1). Considering the comparably short life span of the animals and since it was found that exercising at later ages did not reveal beneficial effects in wild type flies (Piazza et al., 2009), we decided to expose flies to daily training periods of 30 min at a young age rather than during their entire life span. A gentle activity induction protocol consisting of 12 consecutive days of daily exercise during the time of siesta sleep was found to be most effective in young Aβ42-expressing flies shifting survival time back to a comparable niveau observed in control groups (Figure 6).

Monitoring animals during daily exercise units revealed that not all rotations of our device resulted in a locomotor response of each fly, likely due to the fact that test tubes were only slowly tilted by an angle of *φ* = 30°. In case of *elav-GAL4* control strain only 9.4% of stimulations could provoke locomotor activity indicating that efficiency of activity induction is also dependent on intrinsic genetic properties of the used fly strains. Thus, both negative geotaxis and the animals’ innate response to the movement impulse are crucial to successfully induce locomotor activity in *Drosophila*. Furthermore, it was noticed that Aβ42-expressing flies were more accessible to activity induction compared to wild type as well as GAL4 and UAS control strains and reacted to more activity stimulations in average (Figure 2I). Pan-neuronal expression of Aβ42 is likely to cause alterations in the perception of the rocking impulse during activity induction which could be cause for a reinforced response to gravity stimuli. Further, AD flies displayed signs of hyperactivity in terms of increased locomotor activity in young flies and arrhythmic spikes of activity in the night (Figure 2D). Similarly to our findings, hyperactivity and progressive loss of circadian rhythms was reported in *Drosophila* models of Alzheimer’s disease (Chen et al., 2014; Zhang et al., 2015). Altogether gentle exercising did not significantly increase overall locomotor activity and even partially reduced activity in control groups (Supplementary Figures S2A–C, S3A). This can be explained first, by the low dosage of exercise (in order to not to evoke stress in Aβ42-expressing flies) and second, by prolonged resting phases observed after exercising and indicating a compensation of enforced activity. Exercising wild type and control flies were less locomotor active subsequently to activity induction when compared to non-exercising animals (Figures 3A,C,E). Further, quantification of sleep patterns suggests elevated resting in the evening as an instantaneous effect of exercising during time of siesta sleep (Figures 4A–C). The dosage of induced activity seemed too low to evoke long-term changes in behavioral or survival in wild type flies whereas AD-flies were more responsive to exercising. The data suggests that longer or more frequent exercise units are required to intensify the effect in wild type strains while a higher training dosage turned out to be deleterious in Aβ42-expressing flies.

Interestingly, exercising in AD flies was not accompanied by decreased locomotor activity (Figure 3G) and subsequent resting (Figure 4D). Analysis of sleep patterns in Aβ42-expressing flies showed that overall sleep quantity is not affected during the exercise-phase (day 1–12) but activity induction rather led to distinct shifts in sleep distribution. Aβ42-expressing flies displayed significantly more night time sleep and less resting phases in the morning instead of immediately compensating enforced activity after exercising. Since sleep during the night was found to be pathologically reduced in Aβ42-expressing flies in comparison to all control groups (Figures 4A–D), it is conceivable that exercising can partially reconstitute sleep deficits in this fruit fly model of AD. Disturbances of nocturnal sleep with progressing severity have also been reported in AD patients in the course of neurodegeneration (Vitiello and Prinz, 1989) and behavioral approaches could be shown to sustain the treatment of sleep disorders in dementia patients (McCurry et al., 2004).

To further investigate the effect of exercise on sleep phenotypes in Aβ42-expressing flies, episodes of fragmented locomotor activity and sleep were compared between genotypes and treatment groups. Fragmented sleep, for example, is linked to higher risk of AD and cognitive dysfunction in humans (Lim et al., 2013). The depiction of actograms indicated that resting phases in Aβ42-expressing flies were frequently interrupted by spikes of activity, predominantly during the night. Consequently, quantity of fragmented sleep phases was elevated throughout the life span in comparison to control animals (Figures 5G–I). Besides ameliorating deficits in night time sleep our results further demonstrated that exercising can also counteract elevated sleep fragmentation in Aβ42-expressing flies. First, actograms of exercising AD flies show noticeably fewer spikes of activity (Figure 2H) and second, episodes of fragmented sleep were continuously reduced during activity induction as well as in the post-exercise phase (Figure 7A).

Aβ42 deposition in the adult fly brain and mRNA content of *Aβ42* was unaltered by exercise. Although *Aβ42* expression was tendentially decreased (Figure 7B) and the occurrence of Aβ42 containing aggregates was more variable in exercising animals (Figure 7D) there were no significant changes in overall quantity. Exercise induced effects might be differently pronounced in individual animals which could explain only slight changes on expression level and higher variations regarding the formation of aggregates. These results are in line with rodent studies showing that access to running-wheel did not change neuropathological parameters, but reduced the amount of behavioral phenotypes like stereotypic behavior in the TgCRND8 mouse model of AD (Richter et al., 2008). In APP23 transgenic mice carrying another mutation of the gene encoding amyloid precursor protein (APP), wheel running has been shown to reduce the expression of *APP* mRNA (Wolf et al., 2006), and attenuate alterations in Aβ processing (Adlard et al., 2005). In transgenic mouse models of AD that present extensive neurofibrillary tau pathologies treadmill running was also shown to reduce tau phosphorylation (Leem et al., 2009). Although results in rodents are mixed due to the use of different exercise protocols and variant animal characteristics, there is overall some indication that running may be neuroprotective at the molecular level in animal models of AD.

Although one might argue that flies display a number of disadvantages, disparate anatomical situation and different locomotor behavior, it is beyond controversy that several behavioral paradigms may be also applied to flies (open field, social isolation, drug addiction; Meehan and Wilson, 1987; Wolf et al., 2002; Martin, 2004; Joiner et al., 2006; Simon et al., 2012). Despite the obvious anatomical differences between rodent models and invertebrate models like the fly, it is well known that the majority of genetic pathways are conserved and prone to investigation. Thus, the fly may be a good model for exercise and investigation of the underlying molecular mechanisms in the normal and diseased human brain.

Here we demonstrate that our design of the “swing boat” device provides a novel alternative to induce and study physical activity in adult *Drosophila melanogaster*. It stands out by a high degree of automation, objectivity and standardization when compared to other existing, conventional approaches. This exercise device facilitates investigation of a broad range of different disease models on behavioral and molecular level. In addition to simple acquisition and processing of activity data, future studies may include identification of molecular pathways, detection of functional interactions using large scale RNA_i_, or overexpression experiments *in vivo*. In consistence with previous findings in rodent models of AD (Adlard et al., 2005; Wolf et al., 2006; Leem et al., 2009) the application of our methodology showed that exercising can partially remediate Aβ42-induced phenotypes in adult *Drosophila melanogaster*. Attenuation of behavioral abnormalities regarding survival and sleep indicates gentle physical activation to be beneficial to counteract neurodegenerative processes in flies.

## Author Contributions

JB, F-JL, OA, DR, WP and AJ: substantial contributions to the conception or design of the work. JB and F-JL: substantial contributions to acquisition or analysis of data for the work. JB, F-JL, OA, DR, WP and AJ: drafting the work or revising it critically for important intellectual content. JB, F-JL, OA, DR, WP and AJ: final approval of the version to be published. JB, F-JL, OA, DR, WP and AJ: agreement to be accountable for all aspects of the work in ensuring that questions related to the accuracy or integrity of any part of the work are appropriately investigated and resolved.

## Conflict of Interest Statement

The authors declare that the research was conducted in the absence of any commercial or financial relationships that could be construed as a potential conflict of interest.

## Footnotes

^1^http://imagej.nih.gov/ij/
