# Supplementary material for: Swing Boat: Inducing and Recording Locomotor Activity in a Drosophila melanogaster Model of Alzheimer’s Disease
Source: Front Behav Neurosci. 2017 Aug 30;11:159. doi: 10.3389/fnbeh.2017.00159 (PMC5582087; doi:10.3389/fnbeh.2017.00159)
Supplement: Supplementary file 1 [file Presentation_1.pdf]

frontiers

Supplementary Material

Swing boat: Inducing and Recording Locomotor Activity in a *Drosophila melanogaster* Model of Alzheimer's Disease

Johannes Berlandi* & Fang-Ju Lin, Oliver Ambrée, Dirk Rieger, Werner Paulus, Astrid Jeibmann

*** Correspondence:** Johannes Berlandi, M.Sc.: johannes.berlandi@ukmuenster.de

1 Supplementary Figures

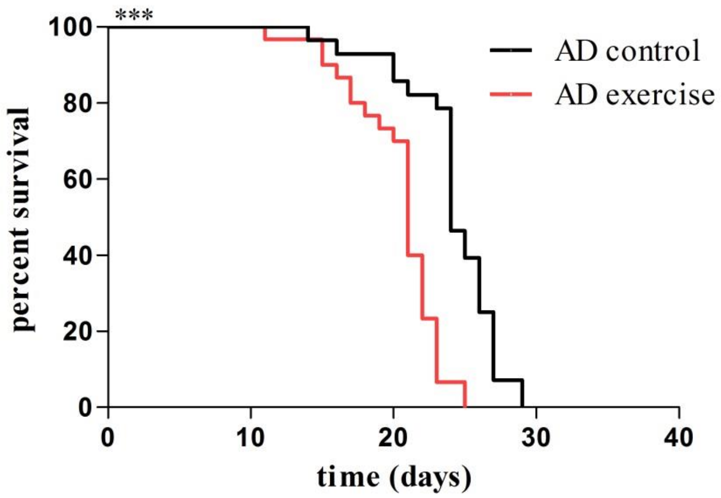

Supplementary Figure 1. Kaplan–Meier plot shows survival rates of exercising Aβ42-expressing flies compared to non-exercising control group. Activity induction was carried out as long as animals in exercising group were alive. Median survival time of exercising AD flies (21 days) was shorter compared to non-exercising AD flies (24 days)($^{***}p<0.001$).

UAS-Aβ42/+ ***elav-GAL4*** **AD**

Activity: control vs. exercise

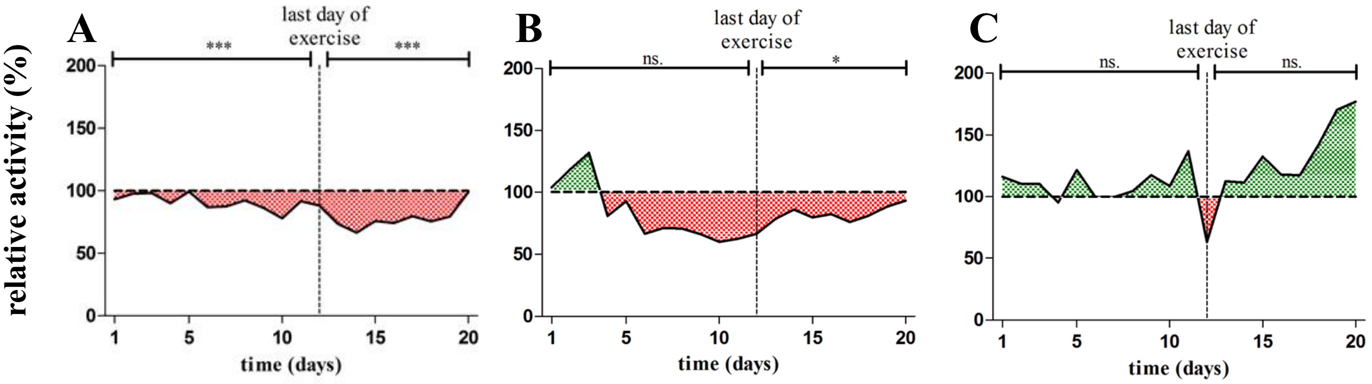

Sleep: control vs. exercise

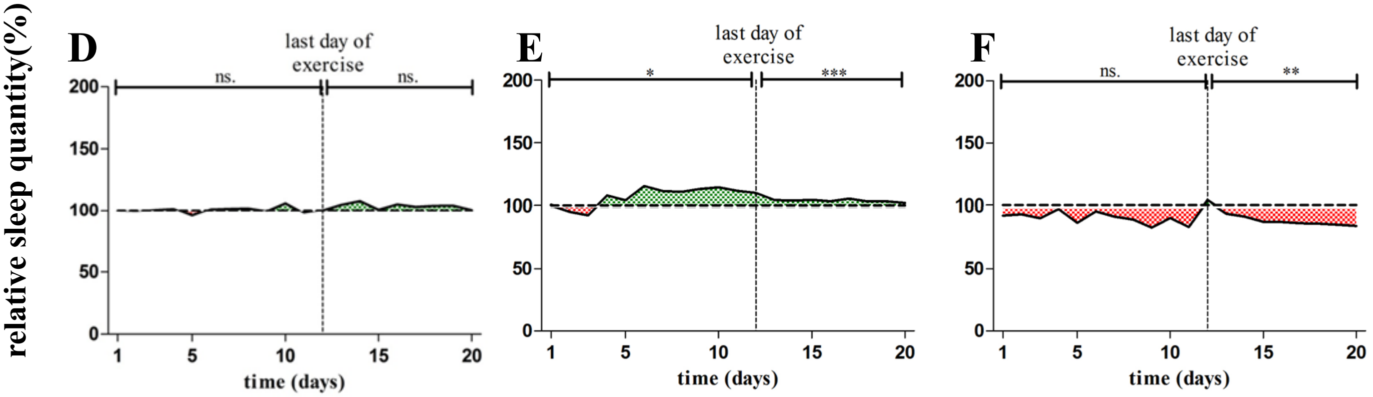

Fragmented Sleep: control vs. exercise

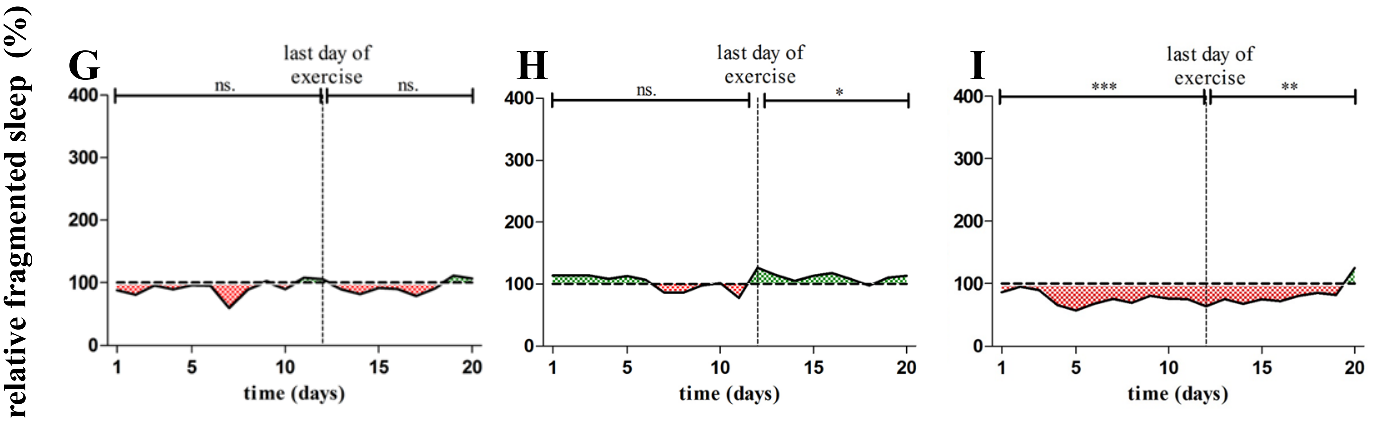

Supplementary Figure 2. Relative activity, sleep and fragmented sleep of exercising *UAS-Aβ42/+*, *elav-GAL4* and AD flies was assessed by normalization to non-exercising control groups. *Drosophila* Activity Monitors were loaded with a total number of 32 animals per genotype. Vertical line at day 12 marks the last day of exercise. Total activity, overall sleep and fragmented sleep were averaged for each day and displayed over a period of 20 days with regard to non-exercising control groups. Niveau of activity, sleep and quantity of fragmented sleep of non-exercising controls is considered as 100%.

Exercising *UAS-Aβ42/+* flies displayed reduced locomotor activity ($^{***}p<0.01$) over a period of 20 days of data acquisition (A). There were no alterations regarding sleep and fragmented sleep quantity following activity induction in *UAS-Aβ42/+* flies (D,G).

Locomotor activity of exercising *elav-GAL4* flies was reduced after completing exercise protocol (day 13-20; $^{*}p<0.05$)(B). Duration of daily sleep was increased in exercising *elav-GAL4* flies from day 1-12 ($^{*}p<0.05$) as well as from day 13-20 (E). Short term sleep of exercising *elav-GAL4* flies was increased after (day 13-20; $^{*}p<0.05$) activity induction compared to non-exercising control (H).

Exercising had no effect on activity of AD flies (C). Exercising reduced sleep in the post-exercise phase (day13-20)($^{**}p<0.01$)(F) as well as decreased fragmented sleep from day 1-12 ($^{***}p<0.01$) and from day 13-20 ($^{**}p<0.01$)(I) in exercising AD flies.

Oregon-R: control vs. exercise

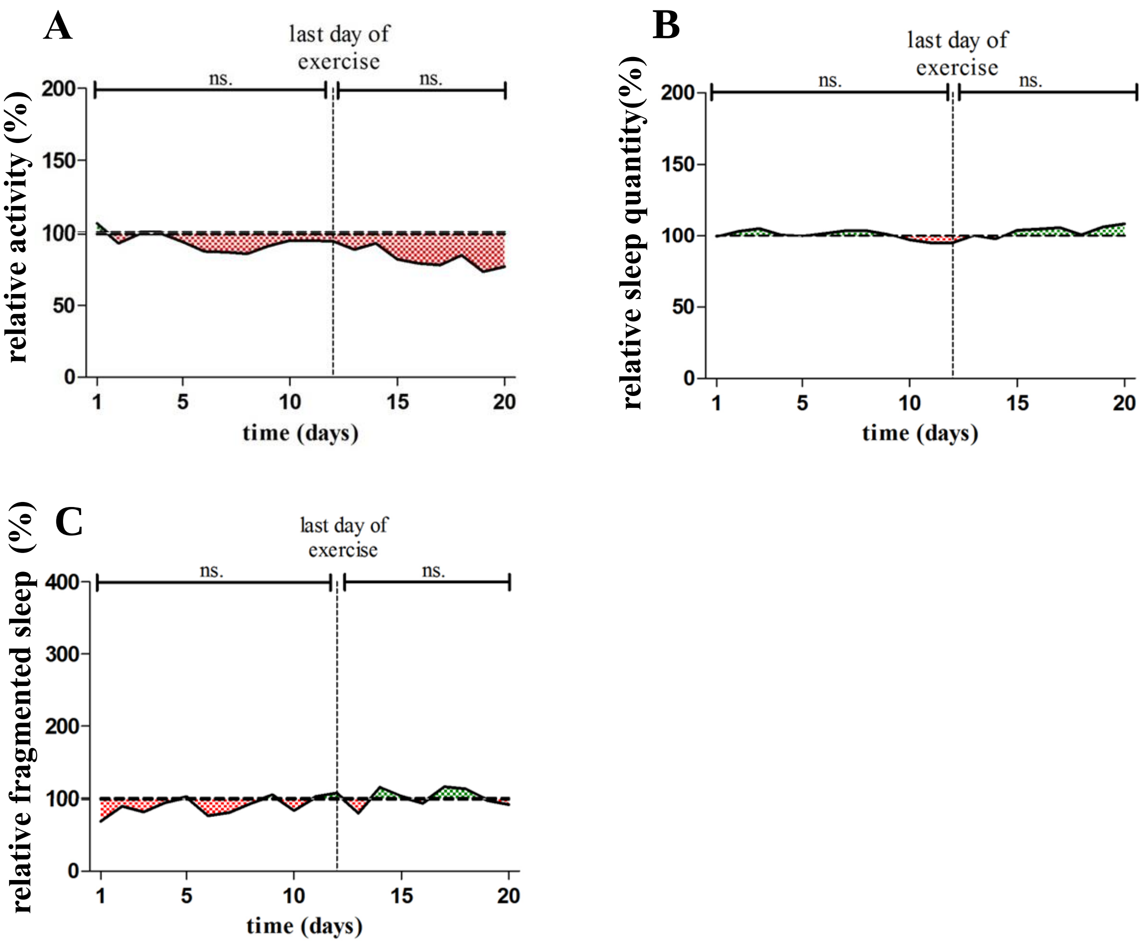

Supplementary Figure 3. Relative activity, sleep and fragmented sleep of exercising Oregon-R flies was assessed by normalization to non-exercising control group. *Drosophila* Activity Monitors were loaded with a total number of 32 animals per genotype. Vertical line at day 12 marks the last day of exercise. Total activity, overall sleep and fragmented sleep were averaged for each day and displayed over a period of 20 days with regard to non-exercising control group. Niveau of activity, sleep and quantity of fragmented sleep of non-exercising control is considered as 100%.
Exercising Oregon-R flies displayed no significant changes in locomotor activity, sleep or fragmented sleep over 20 days of data acquisition (A,B,C).
